# The effect of a patient-oriented treatment decision aid for risk factor management in patients with diabetes (PORTDA-diab): study protocol for a randomised controlled trial

**DOI:** 10.1186/1745-6215-13-219

**Published:** 2012-11-21

**Authors:** Petra Denig, Mathijs Dun, Jan Schuling, Flora M Haaijer-Ruskamp, Jaco Voorham

**Affiliations:** 1 Department of Clinical Pharmacology, University of Groningen, University Medical Center Groningen, Groningen, The Netherlands; 2 Research Institute SHARE of the Graduate School of Medical Sciences, University of Groningen, University Medical Center Groningen, Groningen, The Netherlands; 3 Department of General Practice, University of Groningen, University Medical Center Groningen, Groningen, The Netherlands

**Keywords:** Cardiovascular diseases, Diabetes mellitus, Type 2, Risk assessment, Decision-making, Electronic health records, Primary healthcare, Choice behaviour, Decision support systems, Patient participation, Research design

## Abstract

**Background:**

To improve risk factor management in diabetes, we need to support effective interactions between patients and healthcare providers. Our aim is to develop and evaluate a treatment decision aid that offers personalised information on treatment options and outcomes, and is intended to empower patients in taking a proactive role in their disease management. Important features are: (1) involving patients in setting goals together with their provider; (2) encourage them to prioritise on treatments that maximise relevant outcomes; and (3) integration of the decision aid in the practice setting and workflow. As secondary aim, we want to evaluate the impact of different presentation formats, and learn more from the experiences of the healthcare providers and patients with the decision aid.

**Methods and design:**

We will conduct a randomised trial comparing four formats of the decision aid in a 2×2 factorial design with a control group. Patients with type 2 diabetes managed in 18 to 20 primary care practices in The Netherlands will be recruited. Excluded are patients with a recent myocardial infarction, stroke, heart failure, angina pectoris, terminal illness, cognitive deficits, >65 years at diagnosis, or not able to read Dutch. The decision aid is offered to the patients immediately before their quarterly practice consultation. The same decision information will be available to the healthcare provider for use during consultation. In addition, the providers receive a set of treatment cards, which they can use to discuss the benefits and risks of different options. Patients in the control group will receive care as usual. We will measure the effect of the intervention on patient empowerment, satisfaction with care, beliefs about medication, negative emotions, health status, prescribed medication, and predicted cardiovascular risk. Data will be collected with questionnaires and automated extraction from medical records in 6 months before and after the intervention.

**Discussion:**

This decision aid is innovative in supporting patients and their healthcare providers to make shared decisions about multiple treatments, using the patient’s data from electronic medical records. The results can contribute to the further development and implementation of electronic decision support tools for the management of chronic diseases.

**Trial registration:**

Dutch Trial register NTR1942**.**

## Background

Multifactorial treatment is recommended for patients with type 2 diabetes mellitus to prevent and manage cardiovascular and renal complications [1]. This implies that in addition to glycemic control, strict management of blood pressure, lipid levels, albuminuria, body weight, as well as smoking cessation are considered important. Despite improvements observed in many processes of diabetes care, risk factor control in these patients remains suboptimal [2,3]. Active patient involvement is an important element to achieve improvements in patient outcomes [4,5]. Adequate management of chronic diseases requires not only optimal performance from healthcare providers but also sufficient understanding and motivation of patients to start and sustain complicated medication and lifestyle regimens [6,7]. Most patients with type 2 diabetes have multiple conditions and risk factors which may require prioritisation in treatment plans [8].

An important aspect is how to motivate both patients and professionals to invest in the management of all risk factors [9-11]. Time constraints, lack of support, patients’ motivation and treatment compliance, as well as discrepancies between provider and patient perceptions have been identified as reasons for not providing optimal care in diabetes [10,12,13]. Healthcare providers perceive risk communication as difficult and time-consuming, and want additional tools to help them improve their patients’ understanding of risks [9,14-16]. There is a need for strategies enhancing patient involvement in disease management that are feasible to implement in routine care [7,8,17]. Computer-based support systems and decision aids have been developed to provide risk information and treatment recommendations, and encourage shared decision-making [18,19]. Their benefits in practice, however, appear to be limited [19,20]. They are not widely used [21-23], and several barriers to their implementation have been identified [24-26]. Important factors to success or failure include good integration of the decision aid in the electronic medical record (EMR) system, providing personalised recommendations at the correct moment of decision-making [24,26-28]. Especially, the focus on treatment recommendations and not merely risk assessment presentation appears to be critical. Interventions aimed at improved monitoring of risk factors and calculating risk scores may succeed in improving these processes of care but seldom lead to improved clinical outcomes [29-32]. Interventions that include guidance on treatment options, on the other hand, seem to be more successful in improving intermediate clinical outcomes, such as better glycaemic and blood pressure control [33,34].

### Shared decision-making and risk communication

Many medical decisions are still made with little input from patients. To improve the quality and outcomes of healthcare, shared decision-making is advocated [35]. Diabetes care is complex and the concept of multifactorial risks may not be fully grasped by all patients. Patients seem to focus primarily on blood glucose levels, and may consider blood pressure or lipid control as less important [36,37]. Experiments demonstrated that providing patients with additional information might stimulate physician-patient discussions concerning disease management, and show promise in improving patient outcomes [32,38-41]. Many patients appreciate getting more insight in clinical management issues [42], and report a better understanding of treatment goals after receiving such information [43-45]. This can facilitate patient-provider agreement on treatment goals [8,36,42]. In addition, it is expected to encourage better adherence to treatment [7,15,41]. Engaging patients in their risk assessment and treatment options before consultation can augment more efficient interaction between patients and professionals [15,46]. A proactive role of patients may also result in a more timely adjustment of medication by their physician [38-40]. This last aspect is crucial for achieving better clinical outcomes, since improving only the disease monitoring process without changing treatment decisions and behaviour is not likely to improve clinical outcomes.

### Theoretical framework and development of decision aid

To improve risk factor management in patients with type 2 diabetes, we thus need to stimulate and support effective interactions between patients and healthcare providers. For this, the PORTDA-diab (Patient ORiented Treatment Decision Aid for diabetes) project is set up. In this project, primary care practices will use a novel treatment decision aid focusing on shared goal-setting and decision-making. The aid is to be used before and during consultation by patients and their healthcare providers. This approach will have the potential to address several elements considered important for productive interactions as proposed in the chronic care model and the model for shared decision-making [47,48]. These elements are: (1) clinical information system that facilitates patient-provider conversations; (2) decision support that promotes joint, well-informed goal setting and treatment decisions; (3) proactive healthcare provider and patient, and (4) self-management support through patient empowerment and motivational counselling.

There are many decision aids for patients to support their self-management of diabetes or cardiovascular risk factors but only a few incorporate medication treatment decisions [19,49]. There is debate about the best way to inform patients regarding the benefits and risks of treatment options, and engage them in setting treatment goals [15,50]. So far, two types of treatment decision aids for patients with diabetes or cardiovascular risks have been tested in practice [41,44,51]. The first type includes the ‘diabetes medication choice’ and the ‘statin choice’ decision aids, which are intended to be used during consultation and encourage patients to consider and voice their views about medication options to the clinician [44,51-53]. They were found to be effective in involving patients in the decision-making process but did not clearly improve adherence or clinical outcomes [44,51,54]. They are paper-based to make them usable in various consultation settings. The disadvantage of this paper-based approach is that it impedes automated tailoring to the patient’s medical history or situation. The other tested treatment decision aid is a tailored computerised decision aid where patients can enter clinical data, assess their cardiovascular risk, and weigh their preferences for one or more treatment options [55]. The user may examine interactively the effect of one or more therapies and calculate adjusted risks for starting aspirin, statins, antihypertensives, and smoking cessation. This decision aid has been tested in a small study showing some potential for reducing cardiovascular risk [41]. It is, however, not integrated in the EMR system used by healthcare providers, which means that patients themselves must provide the information to the aid.

Building on this existing knowledge and experience, the PORTDA-diab decision aid was developed to include the following key features:

• tailors the information on treatment goals and options to the individual patient,

• addresses treatment decisions for multiple risk factors,

• presents pros and cons of all treatment options, including doing nothing,

• facilitates comparisons across options,

• uses natural frequencies for outcome probabilities and combines graphs and text with negative and positive framing,

• asks patients to think about the treatment options,

• is used before and during consultation with the healthcare provider,

• provides support at the correct moment for decision-making,

• places the specific medication choice within the consultation,

• is integrated in the electronic medical record system, making full use of available information while allowing for additional data entry or correction,

• fits in the normal workflow of diabetes consultations in primary care practice,

• is consistent with the evidence reflected in clinical practice guidelines.

A step-wise development process was followed, as recommended to enhance the quality of the decision aid [56]. First, an inventory was made of what information patients and providers need for making treatment decisions. Next, the decision aid was reviewed in an interactive session with an expert panel of six patients with diabetes. Several changes were made concerning the information included and comparison with a reference group, the inclusion of probing questions, and the graphical and textual presentation. The revised decision aid was further amended and approved by these subjects through individual exchange. In addition, the aid was reviewed by an independent expert on decision aids for shared decision-making, and a textual review was conducted by a master in Dutch linguistics. Two of the researchers (PD, MD) not involved in the development of the underlying algorithms subsequently tested the content validity of the personalised information by entering numerous combinations of input data and reviewing the output. Finally, the usability of the PORTDA-diab was field tested in 10 consultations with diabetes patients and their healthcare providers in three different practice settings. This resulted in several changes to simplify navigation of the computer program. A few technical issues were solved after this testing to ensure good performance of the decision aid in all participating practices, which use different EMR systems.

### Objectives

The aim of this study is to evaluate positive and negative effects of the novel patient-oriented treatment decision aid (PORTDA-diab) for diabetes patients in primary care. As secondary aims, we want to evaluate the impact of different presentation formats, and learn more from the experiences of healthcare providers and patients when using the PORTDA-diab in daily practice.

### Research questions

1. What is the effect of the PORTDA-diab compared to usual care on:

a) patient empowerment (primary outcome)

b) beliefs about treatment options

c) negative emotions

d) satisfaction with care

e) perceived health status

f) treatment decisions

g) intermediate treatment outcomes and predicted coronary heart disease (CHD) risk

2. To what extent is the effect modified by the presentation medium or format:

a) comparing a paper-based with a computer version

b) comparing a short version presenting treatment effects on CHD risk with an extended version presenting effects also on other outcomes which can be of concern to patients

3. To what extent do healthcare providers and patients adhere to the intended use of the decision aid, and what are their experiences with using it in practice?

## Methods

### Design

**Figure 1:**
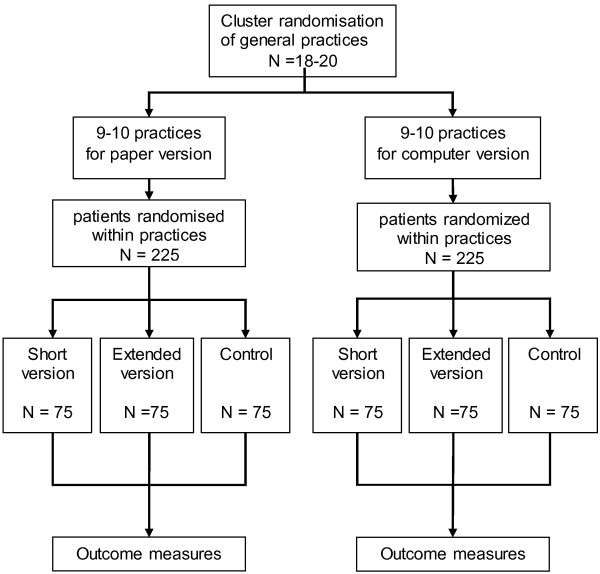
Flowchart of the PORTDA-diab study.

We will evaluate the PORTDA-diab in a randomised pre-post intervention trial using a 2×2 factorial intervention design with a control group (see flowchart in Figure 1). The factorial design is considered the most efficient method to study factors which may influence the outcomes. The inclusion of a control group receiving usual care but undergoing pre-post intervention measurement will control for Hawthorne effects as well as for independent effects or changes in the study population.

### Study setting and participants

We will test the intervention in routine primary care settings in The Netherlands. Primary care practices will be recruited in the Groningen region where a diabetes disease management program has been implemented. Most practices have a practice nurse who conducts quarterly consultations with the diabetes patients. In The Netherlands, it is common that primary care physicians delegate these tasks of chronic care. Practice nurses are trained to conduct practice examinations, risk assessments, patient education, and counselling. They can make medication treatment changes which have to be approved by the physician. To ensure a minimum level of communication skills training in all practices and increase the study participation rate, providers are offered a training session in motivational interviewing before the study starts. In case they already have followed such a course, they are offered €250 as alternative compensation for participation. In addition, all participants will receive a 2-h training in risk communication and an instruction video with simulated consultations showing ‘good and bad examples’ of applying four basic principles of risk communication, that is, to use natural frequencies, use positive and negative phrasing, explicit uncertainty, and be open and refrain from imposing options. All practices use electronic medical record systems supporting structured care protocols. Furthermore, all practices receive yearly performance reports as part of the regional monitoring of diabetes care. We will thus evaluate the additional effect of using the PORTDA-diab over the current disease management program.

Eligible patients include people with type 2 diabetes managed in primary care. Excluded are patients who had a myocardial infarction within the preceding year, experienced a stroke, suffer from heart failure, angina pectoris, or have a terminal illness, and patients who were above 65 years of age at diagnosis, because the calculated risks and treatment goals in these patients are not sufficiently evidence based. After identification of eligible patients in the electronic medical record system, the healthcare providers will confirm that the selected patients satisfy these criteria. In addition, they will exclude patients with dementia or cognitive deficits, who are blind or not able to read Dutch, since such patients are not expected to benefit from this type of intervention. Practices will recruit patients by distributing information packages containing an invitation letter, information about the project, and an informed consent form. Patients can contact the study coordinator for additional information about the study or an independent general practitioner to discuss questions regarding participation. Patients will be offered €10 in compensation for time spent to fill in the study questionnaires. Invitation letters will be followed up by telephone contact by the research team when needed.

### Treatment allocation

**Figure 2:**
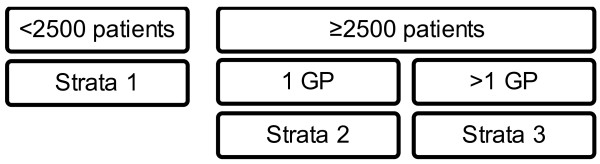
Practice randomisation scheme.

For pragmatic reasons, participating practices will be randomly allocated to the paper-based or computer version. This randomisation will be stratified by practice size (below or above 2,500 patients) and organisation (single or more primary care physicians), using a 1:1 computer-generated allocation sequence within the strata (Figure 2).

Within each practice, consenting patients will be randomised to receiving: (1) the PORTDA-diab presenting only CHD outcomes; (2) the PORTDA-diab presenting additional outcomes; or (3) the control group, using a blockwise scheme to conceal the allocation process for the healthcare provider. Although this design of randomising patients within practices has the disadvantage of potential contamination at provider level, it ensures that variations in communication skills and practice organisation are balanced between the intervention and control group. In addition, low participation and high drop-out rates can be expected when practices are randomised to a non-intervention control arm. It is not possible to use the PORTDA-diab for other patients than the intervention patients. It is, however, possible that providers will use some of the aspects learned from working with the novel approach in their usual care.

### Intervention

The intervention consists of: (1) PORTDA-diab software installed on a computer linked to the electronic medical record system enabling up-to-date data retrieval of the patient’s clinical situation and treatment status; (2) a set of treatment cards which can be used during consultation, summarising positive and side effects of available treatment options; and (3) a short instruction protocol for the healthcare provider about the decision aid and treatment cards (Table 1). The decision aid offers personalised information on treatment goals and options to patients immediately before they consult their healthcare provider. The same information will be available to the healthcare provider for further use during consultation. In practices randomised to the computer version, PORTDA-diab will be installed on a 17 inch laptop or workstation that can be used by the patients. In practices randomised to the paper-based version, a printed version of the personalised information generated by the PORTDA-diab software will be offered to the patients at the practice. Intervention patients will be asked to come to the practice 15 min in advance to go through this additional information. All intervention patients should receive the printed version at the end of the consultation. The short version of the PORTDA-diab presents treatment effects on reducing the risk of getting a myocardial infarction, and comprises five pages or four screens. The extended version covers seven pages or eight screens, and presents treatment effects also on other complications which affect functional activities that are of concern to patients, that is, the risk of getting a stroke, amputation, blindness, and renal failure [9,57,58].

**Table 1:** Instruction protocol for using the PORTDA-diab and treatment cards

	
*Before the consultation*	
· Start PORTDA software and retrieve information for scheduled patients	
· Check whether the relevant patient’s data are available and up-to-date; add or correct data when needed	
· Print the information	
*During the consultation*	
· Start with open question on the patient’s opinion about the information	
· Ask about emotions and/or cognitions related to the information	
· Explore whether the patients already thought about consequences or goals	
· Support patient in thinking about treatment goals and options	
· Ask patient for preferences regarding risks to target (first) and options	
· Explicit treatment options where needed, and use treatment cards when appropriate	
· Do not force a decision but offer the option of a next consultation when needed	
· Ask whether the patients has other matters to discuss	
· Conclude with clear action points (who will do what)	
· Give paper version of the personalised information to the patient	

**Figure 3:**
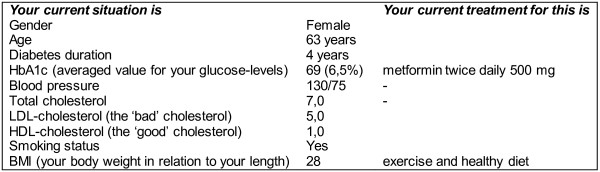
**Patient’s current situation and treatment status. **Brief overview of information collected from the electronic medical record showing the patient’s current situation and treatment.

**Figure 4:**
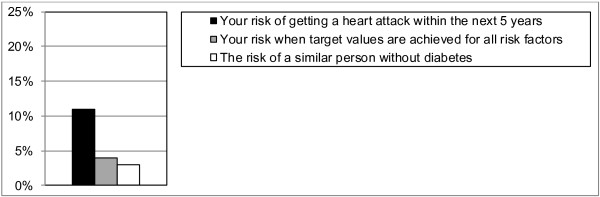
**Bar graph showing the patient’s risk of getting a myocardial infraction. **Shown are the patient’s current 5-year risk, the expected risk when goals are achieved for all risk factors, and the risk of a similar person without diabetes.

**Figure 5:**
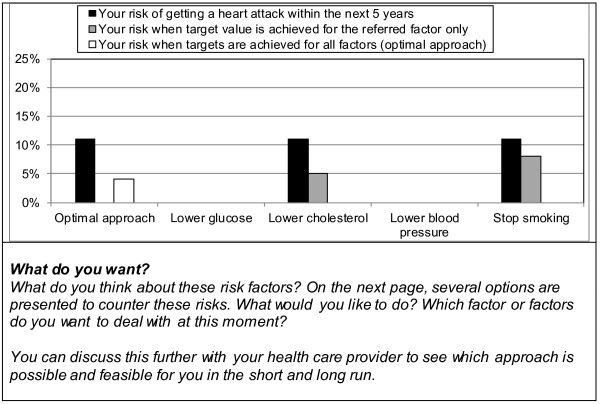
**Bar graph showing the patient’s possible risk reductions for each risk factor with accompanying questions. **Shown are the patient’s possible risk reduction when all or each of the separate risk factors would reach the recommended target values. Below the graph the accompanying questions asked in the decision aid are presented.

The information presented will be generated automatically integrating routinely registered information from the electronic medical records as well as evidence-based information on diabetes treatment and outcomes as summarised in the Dutch Primary Care Guidelines on type 2 Diabetes [59]. The patient data are extracted real time from the medical records but can be corrected or completed by the provider when needed. The information starts with a summary of the patient’s current situation and treatment (Figure 3). The patient’s risk information is presented using bar graphs with simple explanations of critical treatment goals and treatment options [60]. Using the most recent information on the patient’s risk factor levels and the UKPDS risk engine algorithm [61], the patient’s overall risk of getting a myocardial infarct within 5 years is calculated. This overall risk will be presented in relation to potential improvements and to a reference group (Figure 4). Such a reference is important since people can misperceive their initial risk. The patient’s current risk is thus compared to his/her expected risk when achieving the recommended treatment goals for all risk factors (‘optimal treatment’). At the same time, it is compared to the risk of a patient with similar patient characteristics but without diabetes, as calculated by the Framingham risk score [62]. Should the Framingham risk score be higher than the ‘optimal treatment’ risk, the former will be equated to the latter. Next, the impact of achieving recommended goals for separate risk factors will be presented together with possible treatment options. Only risk reductions are presented for risk factors that are not yet at target level. These target levels are derived from the Dutch Primacy Care Guidelines, being <53 mmol/mol (<7%) for HbA1c, <140 mmHg for systolic blood pressure, <2.5 mmol/L for LDL-cholesterol, and not smoking [59]. Patients are explicitly encouraged to think about which of the risk factors they prefer to address first. This will enable prioritisation that may be needed when several changes in treatment are required. In the short version, only the impact on the risk of getting a myocardial infarction is presented (Figure 5). In the extended version, Figure 5 is replicated presenting the impact on the other outcomes [63]. For myocardial infarction and stroke, bars will be visible only if controlling the corresponding risk factor will lead to an absolute risk reduction of 1% or more. For amputation, blindness, and renal failure, bars will be visible at or above an absolute risk reduction of 0.1%. These cut-points are chosen because patients in the pilot phase mentioned that smaller reductions were not meaningful or relevant for them.

### Treatment options and treatment cards

**Figure 6:**
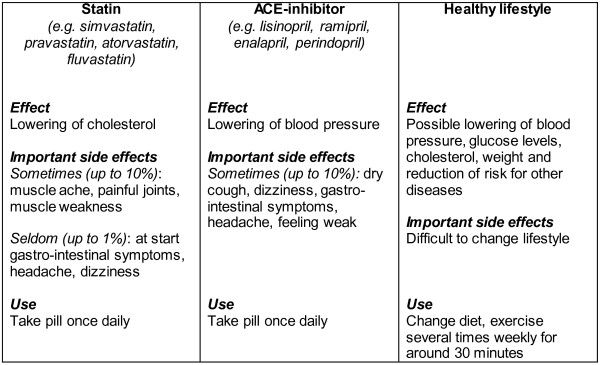
Example of treatment cards.

On the final page or screen, the treatment options are summarised for those risk factors not yet at target level. Both lifestyle and medication options can be presented. The medication options are tailored to whether or not the patient is already treated and/or is already receiving maximum treatment. Maximum treatment is defined per risk factor as: using insulin with or without additional oral glucose-lowering medication, using three or more antihypertensive drugs, using a statin at maximum dosage. Possible pros and cons of the options are summarised. In addition, the healthcare providers can use a set of treatment cards during consultation to discuss and compare the benefits and risks of specific treatment options (Figure 6).

#### Control group

Patients in the control group will receive care as usual, including any education or information as deemed necessary by the healthcare provider.

### Effect modification

Effects of the decision aid may depend on characteristics of the patients that affect their involvement, understanding, and patient-provider communication about disease management. Therefore, the following characteristics will be included as possible modifying factors: age, gender, educational level, socioeconomic status, duration of diabetes, number and type of risk factors present, co-morbidity, and number of drugs used.

### Outcome measures

In correspondence with the aim of the decision aid, our primary outcome is empowerment of patients for making shared decisions about setting and achieving treatment goals [18]. As secondary outcomes, we include a set of additional patient outcome and process of care measures. These will provide insight into possible related positive and negative effects and a better understanding of how the novel approach may affect diabetes care.

Primary patient outcome:

a) diabetes empowerment (Diabetes Empowerment Scale, subscale ‘Setting and Achieving Goals’ [64])

Secondary patient outcomes:

b) satisfaction with diabetes care (PEQ-D [65])

c) beliefs about medication (BMQ [66])

d) negative emotions (PAID [67])

e) health status (EQ-5D [68])

f) predicted coronary heart disease risk [61])

Secondary process of care outcomes:

g) percentage of patients with (intensified) antihypertensive treatment after insufficiently controlled blood pressure levels

h) percentage of patients with (intensified) glucose-lowering treatment after insufficiently controlled HbA1c-levels

i) percentage of patients treated with lipid-lowering drugs

j) percentage of patients with (micro)albuminuria treated with a RAAS inhibitor

The Diabetes Empowerment Scale consists of three subscales measuring different aspects of patient empowerment: managing psychosocial aspects of diabetes (9 items); assessing dissatisfaction and readiness to change (9 items); and setting and achieving goals (10 items) [64]. It has been translated into various languages, and is considered a suitable tool in evaluating empowerment-based education programmes. The subscale on setting and achieving goals will be included as primary outcome. The Patients’ Evaluation of Quality of Diabetes Care is a 14-item scale assessing patients’ judgements about the quality of their diabetes care, which was found to be suitable for care delivered by physicians as well as nurses [64]. The Beliefs about Medication Questionnaire comprises two sections: the BMQ-Specific, which assesses representations of medication prescribed for personal use, and the BMQ-General, which assesses beliefs about medicines in general [66]. It has been translated into Dutch and used in many studies. The BMQ-Specific assesses beliefs about the necessity of and concerns about prescribed medication. The BMQ-General assesses beliefs that medicines are harmful and overused. The Problem Areas in Diabetes (PAID) is a 20-item questionnaire available in Dutch, assessing diabetes-specific emotional functioning [67]. The EQ-5D measures the general health status on five dimensions: mobility, self-care, daily activities, pain and symptoms, and negative emotions, and by a VAS-scale measuring overall health status on a scale from 0 to 100 [68].

### Process evaluation

In the post-intervention questionnaire, all patients will be asked to complete a checklist, which assesses contamination of control patients and exposure of intervention patients to the intervention. Intervention patients have to rate how easy or difficult they rate the decision aid information on a five-point Likert scale. Patients allocated to the computer version will in addition be asked how easy or difficult they rate its navigation properties. Since they will receive the paper version after the consultation, they will be asked for a comparison of the two versions. Providers’ experiences with the decision aid will be assessed in qualitative interviews using a topic list based on previous assessments regarding the implementation of innovations in practice. To assess adherence to the intended use (treatment fidelity), the actual use of the decision aid before and during consultations will be logged automatically in the computer version. In addition, all providers will be asked to complete a short checklist about the PORTDA-diab and the (shared) decision-making process after each consultation with an intervention patient.

### Data collection

Patient data will be collected with structured questionnaires and automated extraction procedures from the medical records. Patients’ empowerment, beliefs, satisfaction, and perceptions will be measured using mailed surveys that all patients will receive in the month before and 3 to 4 months after a scheduled quarterly consultation for diabetes. Data on actual management and clinical outcomes in the 6 months before and after the intervention period will be collected from the Groningen Initiative to Analyse Type 2 diabetes Treatment (GIANTT) database. This GIANTT database includes longitudinal data collected from primary care medical records using validated automated extraction procedures [69].

### Sample size

Sample size was calculated using the primary patient outcome, that is, change in diabetes empowerment scale (DES). Since there is no published minimum level of change considered to be relevant [70], we used a change of 0.2 in the DES score to estimate the sample size. With an expected range in its baseline value of 3.2 to 4.0, this represents a 5% to 6% change in the DES score. To be able to detect an absolute change of 0.2 in the DES score, a total of 150 patients per study arm is needed to achieve 80% power at 5% significance (standard deviation (sd) =0.62). It is therefore estimated that we will need a sample of 18 to 20 GPs, each with at least 20 to 30 participating diabetes patients. This number is expected to be sufficient to detect differences of 0.36 on the PEQ-D (sd=1.1), 6.5 on the PAID (sd=20.0), 0.26 on the BMQ (sd=0.8), and 5.0 on the UKPDS risk estimated (sd=15.5). These are all conservative sd estimates derived either from the original validation studies or available data on the UKPDS risk scores for the GIANTT population. Primary care practices in the recruitment region have on average around 45 type 2 diabetes patients eligible for this study.

### Statistical analysis

Descriptive statistics will be used to summarise demographic, clinical, and other patient measures. The primary analysis will test pre-post intervention changes in patient outcomes in the combined intervention group as compared to the control group using Student t-test and Wilcoxon’s two-sample statistics. Furthermore, we will examine differences in primary and secondary outcomes stratified by presentation format and medium in each intervention arm. For the process of care outcomes, z-approximation statistics will be used. Data will be analysed on an intention-to-treat basis, and results of two-sided tests between study arms will be regarded significant at *P*<0.05. We will explore the influence of the practice on the outcomes by multilevel modelling. Also, for hypothesis generation, interactions will be tested to identify possible factors at patient level that might modify the effect of the intervention.

### Ethical approval and trial registration

The study will be conducted in accordance with the recommendations provided in the Dutch Code of conduct for health research. The medical ethics committee of the University Medical Centre Groningen approved the study (ARB number NL29042.042.09). The GIANTT project and its data collection and database are registered at the CBP (College Bescherming Persoonsgegevens, number 1250778). The trial is registered at the Dutch Trial register NTR1942 with the acronym PORTDA-diab.

## Discussion

The proposed study focuses on the much-needed translation of evidence-based diabetes care in real world settings [71]. It focuses on patient empowerment and patient-centred shared goal setting, which are considered priority areas for further research. It builds upon previous behavioural and implementation research which identified elements, such as proactive surveillance, patient involvement in setting realistic treatment goals and strategies, outcomes-related processes, and use of clinical information systems to improve the quality of care, as some of the key characteristics of effective diabetes management. There is growing evidence that active patient involvement improves diabetes care but the focus has been mostly on dietary and glucose regulation issues. Little attention has been given to involving patients in the overall treatment decision-making process.

Several complex, multifaceted implementation strategies have shown to improve diabetes care in experimental settings but they are seldom replicated or sustained in daily practice [4,20,72]. Healthcare providers have many competing demands, and especially small healthcare organisations have few resources for conducting complex interventions [72]. Despite positive attitudes that healthcare providers and patients express regarding shared goal-setting and decision-making, they appear to have difficulties to translate these intentions into practice [15,42,45]. Simple tools that can help to structure and prioritise treatment goals and plans are needed. The PORTDA-diab developed in this study can be characterised as a minimal-intensity strategy that requires little extra time to conduct in a normal practice setting. Strategies that can prepare patients better for a consultation on disease management have the potential of being very cost-effective.

The decision aid is developed building on existing knowledge and following most of the recommendations for high quality decision aids [56]. To the best of our knowledge, there have been no rigorous studies evaluating the effects of patient-oriented treatment decision aids that deal with multiple treatment goals and plans for individual patients. Recently, two newly developed decision aids dealing with multifactorial cardiovascular treatment have been pilot tested, showing acceptability and feasibility of using such decision aids in primary care settings [27,28].

The outcome measures in our study have been chosen to provide detailed information on the process and outcomes of the intervention. Changes in patients’ empowerment and perceptions will be measured with structured questionnaires. Data on actual management and clinical outcomes will be assessed up to 6 months after the intervention allowing for delayed actions. The study is not designed or powered to assess any long-term outcomes regarding morbidity or mortality. By inclusion of the predicted absolute coronary heart disease risk as secondary outcome measure, however, we will be able to draw conclusions on potential benefits on long-term outcomes.

There is scarce information on how positive and negative effects of patient-oriented decision aids may differ for different patients [18,73]. Diabetes patients will typically differ in the severity and duration of their disease, the number of co-morbid conditions, and the number of medications they need. Patients may also differ in experiencing negative feelings when confronted with personal risk information. Some patients have trouble with the quantitative scoring of risk and the clinical focus on numerical treatment goals instead of functional goals [8,15,17,42,57,58,74]. Patients can furthermore differ in their preferences and abilities to participate in treatment decision-making [8,58,74], and in their information needs. This study aims to include a heterogeneous group of patients with type 2 diabetes. By examining interaction effects of the intervention in relation to inter-individual differences, the study can provide insights into the utility and feasibility of using this type of decision support in various patient populations. The population is, however, restricted on specific clinical characteristics, such as age at diagnosis (≤65 years), and on cognitive and reading abilities.

The study will specifically address two matters of interest, which can contribute to the further development of interactive (web-based) programs for the management of chronic diseases [75]. Many computer and web-based decision aids are being developed but there are concerns that not all patients may be able to use such systems. This study will provide information on differential effects of using a paper-based as compared to a computer-based decision aid, looking particularly at patient demographics, such as age, gender, educational level, and socioeconomic status. There are also questions regarding reframing messages about benefits of treatment to the perspective of the patient [57,76]. Most patient decision aids formulate outcomes in terms of major clinical endpoints, such as the risk of dying or - in case of cardiovascular risk management - the risk of getting a myocardial infarction. This study will assess what the effects can be of giving additional information on other outcomes that some patients might consider of more importance.

A possible limitation of our study may be selection bias of participating practices. They may represent a group that is more open to shared decision-making. Since this study aims to develop and pilot an innovative strategy, we feel this is not a major drawback. Furthermore, the short duration of the intervention may limit the potential of the decision aid. Although the intervention allows for a follow-up consultation, it is possible that repeated use of the decision aid can enhance its effect. We will be able to use the results from the process evaluation to make adjustments or recommendations for further implementation of treatment decision aids in practice. If this aid is found to be helpful, it can be implemented and tested on a larger scale. The approach can also be translated to other areas of chronic disease management where patients are confronted with multiple treatment goals and plans.

## Trial status

Patient recruitment continued until September 2012. Baseline data collection will be completed in 2012. Outcome data collection will be completed in 2013.

## Abbreviations

BMI: Body mass index; CHD: Coronary heart disease; EMR: Electronic medical record; HbA1c: Glycated haemoglobin; HDL-cholesterol: High density lipoprotein cholesterol; LDL-cholesterol: Low density lipoprotein cholesterol; PORTDA-diab: Patient-oriented treatment decision aid for diabetes.

## Competing interests

The authors declare that they have no competing interests.

## Authors’ contributions

Conception of study and formulation of research questions: PD, JV, and FMH-R. Development and pilot testing of decision aid: PD, JV, JS, and MD. Drafting the manuscript: PD, JV, and MD. Critical revision and final approval: JV, MD, JS, FMH-R, and PD. All authors read and approved the final manuscript.
